# Variations in the Physical Performance of Olympic Boxers over a Four-Day National Qualifying Tournament

**DOI:** 10.3390/sports9050062

**Published:** 2021-05-12

**Authors:** Irineu Loturco, Michael R. McGuigan, Tomás T. Freitas, Chris Bishop, Pedro E. Alcaraz, Felipe Romano, Mateus Alves, Valter P. Reis, Lucas A. Pereira, Emerson Franchini

**Affiliations:** 1 NAR—Nucleus of High Performance in Sport, São Paulo 04753-060, Brazil; tfreitas@ucam.edu (T.T.F.); valterpreis@gmail.com (V.P.R.); lucasa_pereira@outlook.com (L.A.P.); 2 Department of Human Movement Science, Federal University of São Paulo, São Paulo 11015-020, Brazil; 3 Faculty of Life Sciences and Education, University of South Wales, Pontypridd CF037 1DL, Wales, UK; 4 Sports Performance Research Institute New Zealand (SPRINZ), Auckland University of Technology, Auckland 0632, New Zealand; michael.mcguigan@aut.ac.nz; 5 School of Medical and Health Sciences, Edith Cowan University, Perth 6027, Australia; 6 UCAM Research Center for High Performance Sport, Catholic University of Murcia, 30107 Murcia, Spain; palcaraz@ucam.edu; 7 Faculty of Sport Sciences, Catholic University of Murcia, 30107 Murcia, Spain; 8 Faculty of Science and Technology, London Sports Institute, Middlesex University, London NW1 4RL, UK; C.Bishop@mdx.ac.uk; 9 Brazilian Boxing Confederation, São Paulo 05075-010, Brazil; felipecbboxe@gmail.com (F.R.); mateus.alvescbb@gmail.com (M.A.); 10 Martial Arts and Combat Sports Research Group, School of Physical Education and Sport, University of São Paulo, São Paulo 05508-030, Brazil; emersonfranchini@hotmail.com

**Keywords:** combat sports, athletic performance, muscle power, weight loss, boxing

## Abstract

The aim of this study was to examine changes in body mass (BM) and power-related measures in Olympic boxers during an official qualifying boxing tournament. Fourteen elite amateur boxers from the Brazilian National Team (eight men and six women) participated in this study. Athletes performed three fights in four days against the same opponent of the same weight-category. Before and immediately after every fight, BM, countermovement jump (CMJ) height, and power production in the bench-press and half-squat exercises were assessed in the same order and on the same time of the day. A two-way repeated-measures ANOVA with the Bonferroni post-hoc analysis was used to determine the variations between pre- and post-measures. The statistical significance was set as *p* < 0.05. The athletes were able to maintain their baseline weight and physical performance throughout the experimental period, as shown by the lack of significant changes in BM, CMJ height, and upper- and lower-limb power output. Throughout a four-day qualifying tournament, the BM and power-related performance of Olympic boxers were not affected either by match execution or by successive matches. As scoring actions are highly dependent on muscle power, it is likely that these combat athletes are able to maintain optimal levels of performance across consecutive matches.

## 1. Introduction

Olympic boxing is a striking combat sport performed in different weight categories and demanding high levels of power, speed, and aerobic and anaerobic power [1,2]. Unlike other combat sports where each weight category performs on a single day (e.g., karate, judo, and taekwondo), Olympic boxing competitions occur over several days [3]. As boxers commonly adopt rapid weight loss procedures during the week prior to official competitions [3], and a new weigh-in is executed on the same day as each match, maintaining body mass (BM) over the competition is a great challenge for boxers [3,4]. Therefore, boxers need to recover properly from the multiple weigh-ins and matches to enhance the chances of achieving successful performances during competitions.

Both upper- and lower-body power seem to be relevant contributors to punching impact [5,6]. In addition, force- and power-related variables have been shown to be strongly associated with distinct activities during international boxing matches, including the total number of rear-hand hooks (*r* = 0.79 with squat jump height), punches thrown to the body (*r* = 0.73 with countermovement jump height), and the effectiveness of straight rear-hand and head punches (*r* = 0.75, with the rate of force development in the squat jump) [7]. Thus, the assessment of power output is a key element in understanding the relationship between recovery and performance throughout competitions. In combat sports, the measurement of muscle power and metabolic responses during combat is not possible with current technology. Due to this limitation, a series of investigations have been conducted to describe the physical and physiological responses to single and multiple matches in Olympic combat sports, including boxing [8,9], karate [10,11], judo [12,13], taekwondo [14,15], Greco-Roman [16,17], and freestyle wrestling [18,19].

Analyses of entire tournaments are even more difficult, with only one study examining the physiological responses of combat sports athletes during a Greco-Roman Wrestling World Championship [17]. This information would allow coaches and practitioners to make more appropriate decisions about training and recovery practices in the course of official championships. Nonetheless, no studies have simultaneously investigated variations in BM and physical performance during boxing competitions. Therefore, the objective of this study was to analyze the changes in BM and power-related measures that occurred in Olympic boxers across an official qualifying boxing tournament.

## 2. Methods

### 2.1. Participants

Fourteen elite amateur boxers from the Brazilian National Team (8 men (2 welterweight—63 kg; 1 middleweight—75 kg; 2 light heavyweight—81 kg; 2 heavyweight—91 kg; 1 super heavyweight—91 kg) and 6 women (1 flyweight—51 kg; 2 featherweight—57 kg; 1 lightweight—63 kg; 1 welterweight—69 kg; 1 middleweight—75 kg); 24.3 ± 4.3 years; 175.6 ± 9.5 cm; 77.3 ± 16.8 kg) participated in this study. The sample comprised 1 World Champion, 6 Pan-American Games medalists, 1 Youth Olympic Games gold medalist, and 1 Youth Olympic Games bronze medalist, attesting to their high level of competitiveness. The study was approved by the local Ethics Committee and all athletes signed an informed consent form before participating in the study.

### 2.2. Study Design

**Figure 1:**
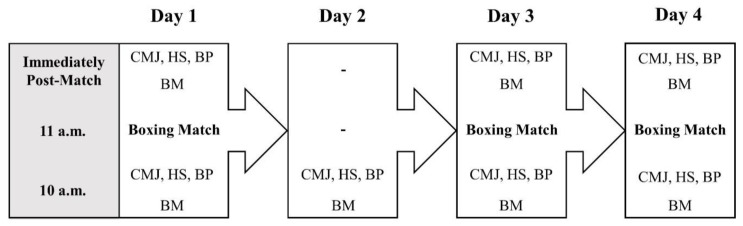
A schematic presentation of the study design. BM: body mass; BP: bench-press; CMJ: countermovement jump; HS: half-squat.

This descriptive study assessed the variations in BM and neuromuscular performance of amateur boxers during a National qualifying tournament. A schematic presentation of the study design is shown in Figure 1. Athletes performed 3 fights in 4 days against the same opponent. The boxer who won more combats (at least 2 out of 3) would gain a place in the Brazilian Olympic Boxing Team (First Team). Before and immediately after every match, BM, countermovement jump (CMJ) height, and maximum power output in the bench-press (BP) and half-squat (HS) exercises were measured in the same order and on the same time of the day. On the day without combats, the physical tests were performed under the same standardized protocol. Before performing the tests, athletes completed a 10 min standardized warm-up, comprising 5 min of running at moderate pace followed by 5 min of dynamic stretching, for both upper- and lower limbs. Prior to the actual evaluations, the athletes performed 5 submaximal trials of each specific test with a 30 s interval between each trial. During the period of the study, the nutritional and sleep habits of the athletes were controlled by the technical staff of the Brazilian National Team.

### 2.3. Procedures

#### 2.3.1. Body Mass

BM was measured using a digital scale with an accuracy of 0.1 kg (Filizola Industry, São Paulo, Brazil) [20]. The measurements were performed prior to the warm-up activity, with athletes barefoot and wearing similar training clothes in all assessments.

#### 2.3.2. Countermovement Jump Test

To perform the CMJ, athletes were instructed to execute a downward movement followed by a complete knee extension. All attempts were executed with the hands placed on the hips. The CMJ was performed on a contact platform (Elite Jump System^®^; S2 Sports, São Paulo, Brazil) and the jump height was calculated based on the flight time. A total of five attempts were allowed, interspersed by 15 s. The best attempt was retained for data analysis.

#### 2.3.3. Bar-Power Outputs in Bench-Press and Half-Squat Exercises

Maximum bar-power outputs were assessed in the BP and HS exercises, performed on a Smith-machine device (Hammer Strength Equipment, Rosemont, IL, USA). Participants were instructed to execute three repetitions at maximal velocity for each load, starting at 30% BM in the BP and 50% BM in the HS. For the BP, athletes were instructed to lower the bar in a controlled manner until the barbell lightly touched their chest and then move the load as fast as possible, without losing contact with the barbell. In the HS, athletes executed knee flexion until the thigh was parallel to the ground, and, after the command to start, subjects were instructed to move the bar as fast as possible, without losing foot contact with the ground, keeping their heels on the floor. In both exercises, a load of 5% BM for the BP and 10% BM for the HS was progressively added for each set until a clear decrement (at least 5%) in mean propulsive power (MPP) was observed [21,22,23]. A 5 min rest period was allowed between sets. To determine the power output, a linear velocity transducer (T-Force, Dynamic Measurement System; Ergotech Consulting S.L., Murcia, Spain) was attached to the barbell and values were automatically collected and calculated by the custom-designed software. The bar position data were sampled at 1000 Hz. The maximum MPP values obtained in each exercise were used for analysis. To account for the differences in the BM of the athletes, values were normalized by dividing the absolute power value by their respective BM (i.e., relative power = W·kg^−1^).

### 2.4. Statistical Analyses

Data are presented as means ± standard deviation (SD). The Shapiro–Wilk test was used to confirm the normality of the data. To examine the differences in athlete performance among the tests executed at the baseline (e.g., at 10 a.m.) on each of the four experimental days, a repeated-measures analysis of variance followed by the Bonferroni post-hoc analysis was conducted. In addition, a two-way ANOVA with repeated measures also followed by the Bonferroni post-hoc analysis was used to analyze the interactions between pre- and post-fight measures on the three fight days (Figure 1). The statistical significance was set as *p* < 0.05. Finally, to determine the magnitude of differences, Cohen’s *d* [24] effect sizes (ES) were calculated and interpreted using the thresholds proposed by Rhea [25] for highly trained individuals, as follows: <0.25, 0.25–0.50, 0.50–1.00, and >1.00 for trivial, small, moderate, and large, respectively. The statistical power was calculated using G*Power software (v. 3.1.9.2), computing the number of subjects, ES, and α values for the different comparisons performed in all tested variables. All measurements used here demonstrated small errors of measurement, as evidenced by their high levels of accuracy and reproducibility (i.e., coefficient of variation <5%, and intraclass correlation coefficient (using an alpha two-way mixed model) >0.90 for all assessments) [26].

## 3. Results

**Figure 2:**
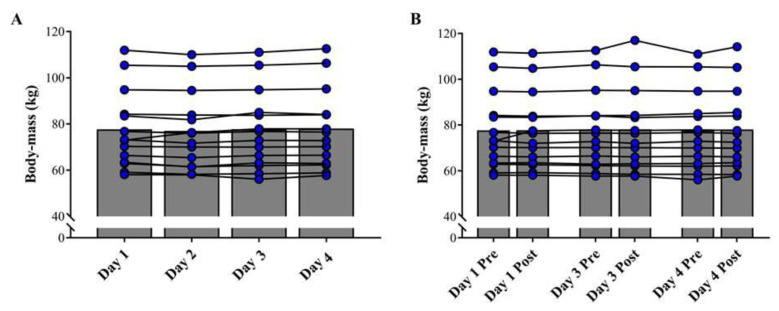
Comparison of baseline values of body-mass on the 4 experimental days (**A**) and between pre- and post-fight assessments (**B**). Symbols represent individual values and bars represent mean results.

**Figure 3:**
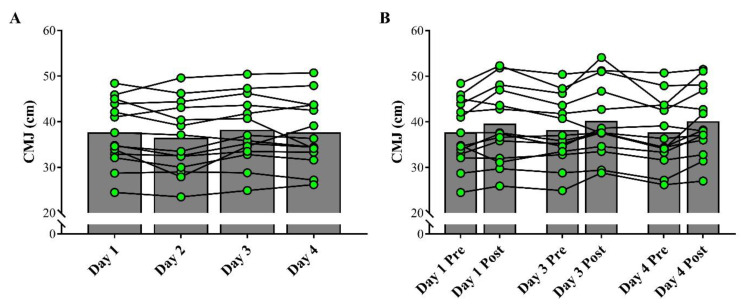
Comparison of baseline values of countermovement jump (CMJ) on the 4 experimental days (**A**) and between pre- and post-fight assessments (**B**). Symbols represent individual values and bars represent mean results.

The statistical power values achieved for the distinct comparisons performed were all >80%. Figure 2A shows the comparison of the baseline values of BM, during the four experimental days, and Figure 2B depicts the comparisons between pre- and post-fight measurements. No significant differences were detected among all tests performed (ES range: 0.01–0.05; *p* > 0.05). Figure 3A shows the comparison of CMJ height, among the baseline assessments on the four days. Figure 3B shows the comparisons of CMJ height between pre- and post-fight assessments completed during the qualifying tournament. Overall, athletes did not show significant changes in the CMJ performance throughout the experimental period (ES range: 0.01–0.22; *p* > 0.05).

**Figure 4:**
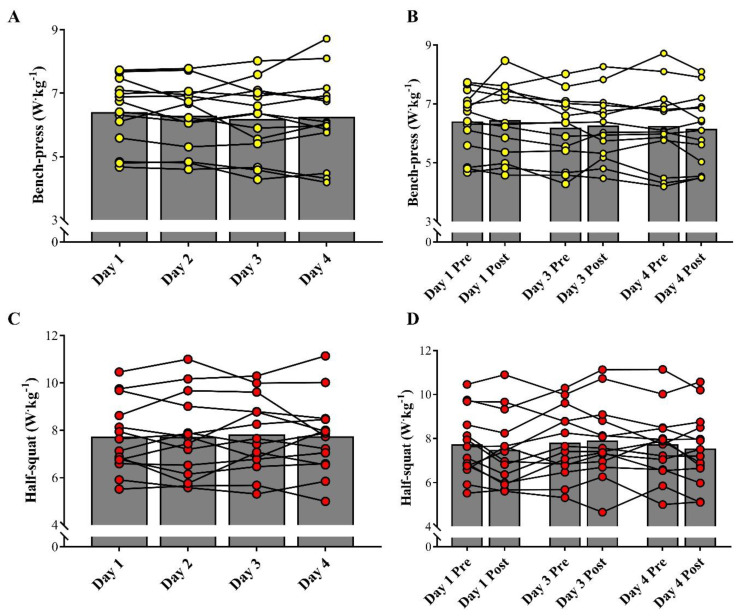
Comparison of baseline values of bench-press and half-squat on the 4 experimental days (**A**,**C**) and between pre- and post-fight assessments (**B**,**D**). Symbols represent individual values and bars represent mean results.

Figure 4A shows the comparison of power output in the BP exercise between the baseline tests, and Figure 4B demonstrates the changes between pre- and post-fight measurements throughout the qualifying tournament. No significant differences were detected among the different measurements (ES range: 0.04–0.18; *p* > 0.05). Figure 4C depicts the comparison of the bar-power output in the HS exercise between the baseline tests, and Figure 4D shows the pre- and post-fight comparisons on the three competitive days. Similarly, athletes did not show significant changes in the HS power throughout the experimental period (ES range 0.01–0.20; *p* > 0.05).

## 4. Discussion

We assessed the variations that occur in the physical performance of elite amateur boxers by comparing pre- and post-fight measures over a four-day qualifying tournament. As expected, the athletes were able to maintain their baseline weight and physical performance throughout the experimental period, as shown by the lack of significant changes in BM, CMJ height, and upper- and lower-limb power output. This is the first study to report these findings in Olympic boxers, who regularly train and compete under these conditions.

As previously mentioned, amateur boxing championships are the only combat competitions where combat athletes are required to weigh-in every day, from the first day of competition, and before successive matches [27]. In general, during national and international amateur boxing events (including Olympic Games and World Boxing Championships), boxers fight four to six times, in periods ranging from four to fourteen days. This competition format has several implications for physical performance and BM control. A previous study reported that 100% of the Brazilian Olympic Team boxers used rapid weight loss procedures before competitions, including exercising wearing sweat suits, restricting liquid intake, and fasting or intermittent fasting [28]. As these rapid weight loss procedures have been reported to negatively affect anaerobic performance when the interval between weigh-in and performance assessment is shorter than 3–4 h [29], boxers need to present fast recovery from their previous match and maintain their BM throughout the tournament, while avoiding decrements in performance. No previous studies have assessed both BM and power output during boxing bouts over several days as in the present study. It seems, however, that high-level Olympic boxers are well-adapted to this format and are able to maintain both their BM and power-related performance in three matches executed in four days. Therefore, it is likely that power capabilities can also be maintained in longer competitions (e.g., 7–12 days), although the maintenance of BM may become more difficult over longer periods, which, in turn, may negatively affect performance.

The power production, especially in the lower extremities, is strongly associated with punching acceleration and impact, for different strokes (i.e., jabs and crosses) and techniques (i.e., punching from fixed- or self-selected distances) [6,30]. Super-elite combat athletes (e.g., a World Karate Champion and an Olympic Boxing Champion) usually present higher levels of JS and BP power than their National Team peers do (i.e., on average, 45 and 12% more power in JS and BP, respectively) [21,31]. Importantly, a recent study revealed that more powerful boxers (in CMJ and SJ tests) were more active and accumulated less stoppage time during matches, also demonstrating the higher effectiveness of head punches (i.e., % of punches that successfully reached the head in relation to total head punches) [7]. Furthermore, in that study, the authors detected a strong correlation between jump height and the total number of body punches (*r* = 0.73) and rear-hand hooks (*r*~0.79) thrown over competitive bouts [7]. In a more applied context, our results might suggest that Olympic boxers can maintain punching power, hand speed, and strike accuracy across consecutive daily matches, at least over short-term tournaments (i.e., 4–5 days). This opens new opportunities for using preparation strategies able to improve performance in the 1–12 h prior to competition (i.e., priming exercises) or even immediately before the combats (i.e., by applying modified warm-up practices including post-activation potentiation techniques) [32,33]. Another possibility could be the utilization of short-term low-volume training schemes throughout longer tournaments (i.e., 2–3 weeks), which have already been shown to produce significant increases in punching impact [23]. As boxers seem to not be affected by neuromuscular fatigue after matches and on subsequent days, coaches may consider incorporating these activities over the course of amateur boxing championships, with the goal of optimizing strength–power capabilities. Nevertheless, we recognize that this is a simple assumption that should be tested in a different study design (i.e., longitudinal intervention).

In summary, we showed that elite amateur boxers are able to maintain their initial BM and levels of upper- and lower-limb power throughout a four-day qualifying tournament. This research is limited by its small sample size and the impossibility of directly measuring punching impact during the fights. Moreover, we did not have access to video-based information, which would allow us to evaluate variations in punching frequency across successive bouts. However, for the first time, we assessed the changes that occur in the physical performance and BM of elite amateur boxers within a real competitive scenario. Future studies are needed to analyze potential alterations in the number and frequency of strokes, as well as the effects of short-term training programs on the strength–power performance of Olympic boxers during actual competitions.

## 5. Conclusions

During a four-day qualifying tournament, the BM and power-related performance of Olympic boxers are not affected either by match execution or by successive matches. As scoring actions are highly dependent on power output, it is likely that these athletes are also able to maintain optimal levels of performance throughout the matches. This suggests that coaches may focus on technical and tactical instructions during the time interval between consecutive matches. Specific warm-up approaches (e.g., strategies including post-activation potentiation techniques), priming exercises, or short-term low-volume training interventions to increase, for example, punching impact should be considered and investigated, as they appear to be effective in this regard.

## Data Availability

All data generated or analysed during this study are included in this published article.
